# Seeing the fields through the weeds: introducing the WeedEco R package for comparing past and present arable farming systems using functional weed ecology

**DOI:** 10.1007/s00334-023-00964-8

**Published:** 2023-10-19

**Authors:** Elizabeth Stroud, Michael Charles, Glynis Jones, John G. Hodgson, Amy Bogaard

**Affiliations:** 1 School of Archaeology, University of Oxford, 1 South Parks Road, Oxford, OX1 3TG UK; 2 Department of Archaeology, University of Sheffield, Minalloy House, 10-16 Regent St, Sheffield City Centre, Sheffield, S1 3NJ UK

**Keywords:** Arable farming regimes, Archaeobotany, Statistical R package, Functional weed ecology

## Abstract

The functional ecology of arable weeds provides a way of comparing present-day and past farming regimes. This paper presents the R package WeedEco, an open-source resource which allows users to compare their archaeobotanical dataset against three previously published arable weed models to understand fertility, disturbance or a combination of both. The package provides functions for data organisation, classification and visualisation, allowing users to enter raw archaeobotanical data, obtain trait values from the functional trait dataset, conduct discriminant analysis and plot the results against the relevant present-day model. Using data from the early medieval site of Stafford in the UK, the paper provides a detailed example of the use of the package, demonstrating its different functions, as well as how the results can be interpreted.

**Supplementary Information:**

The online version contains supplementary material available at 10.1007/s00334-023-00964-8.

## Introduction

The functional ecology of arable weeds offers a means of comparing present-day and past farming regimes, of disentangling the ecological variables that characterise them and of exploring ancient regimes that have no modern analogue (Charles et al. 1997; Jones 2002; Bogaard 2004). Functional ecology identifies a series of traits (or ‘attributes’) that reflect species’ ecological potential in relation to environmental variables such as soil productivity and disturbance (Grime et al. 1988; Grime 2001; Garnier et al. 2016). These traits are morphological or behavioural characteristics that bear a functional relationship to species’ responses to these ecological parameters (Charles et al. 1997; Jones 2002; Bogaard 2004).

In this paper we use a functional trait dataset published by Hodgson et al. (2023) for nearly 1,000 arable weed species occurring in Europe and North Africa, to compare present-day and past weed flora on the basis of these traits. Hodgson et al. (2023) presents the values for traits which were identified in previous studies as useful for discriminating present-day farming regimes that differ primarily in terms of soil productivity and/or disturbance. The weed survey studies undertaken to characterise the present-day regimes in Europe and North Africa have been published over the past decades (Jones et al. 1995, 1999, 2000; Charles et al. 2002; Bogaard et al. 2016, 2018, 2022). Further survey studies are desirable to broaden the range of scenarios and comparisons, including other world regions. The functional trait data can also be used in other, exploratory ways: for example, to assess the relevance of the traits to compositional trends among archaeobotanical samples (Bogaard et al. 2016; Diffey et al. 2020; Stroud et al. 2021), or to characterise the ecology of newly surveyed crop fields (Charles et al. 2002; Bogaard et al. 2018).

Below, first, we introduce three previously published models (Bogaard et al. 2016, 2018, 2022). These compare the functional traits of present-day weed floras with known farming regimes. From these constructed discriminant models the functional traits of past weed floras can be used to predict farming regime. Secondly, to make this methodology freely available, we present the newly created R package WeedEco, which allows users to run these models. The R package WeedEco has been designed for users wishing to compare unknown data against our previously published models (Bogaard et al. 2016, 2018, 2022). It provides functions for data organisation, classification and visualisation.

## The models

Discriminant analysis, a multivariate statistical technique and a form of machine learning, is utilised to identify the best separation between two farming regimes based on the loaded functional ecological traits. Once the equation that best separates the two regimes is found, it can then be used to classify archaeobotanical data as either regime one or regime two. The R package presented below provides the tools needed to take a raw archaeobotanical weed dataset and to conduct linear discriminant analysis to understand where the archaeobotanical samples fall in comparison with the three presented modern models.

Three models are presented here, and while these models have been previously published, this paper provides for the first time the data required to run them (see ESM 1, and data section). For ease of use the different models are referred to as model 1, 2, or 3 based solely on order of publication and are briefly summarised below.

### Model 1: high- versus low-input farming (Asturias and Provence)

In 2016 Bogaard et al. published the results of modern field surveys in Haute Provence, the low input farming regime side of model 1. These data were combined with data from Asturias, Spain which provided the contrasting high input farming regime side of a newly constructed model (Jones et al. 1995; Charles et al. 2002). Bogaard et al. (2016) conducted discriminant analysis of these two datasets, identifying which functional traits best separated the two groups. The resulting discriminant model separated the two regimes based on five functional traits (or attributes): canopy height, canopy diameter, leaf area per node/leaf thickness, mean specific leaf area, and length of flowering period. The results indicated that differences in fertility and disturbance were the driving ecological processes separating the two regimes, with the highly fertile, highly disturbed Asturian plots contrasting with the low fertility, low disturbance Haute Provence fields. This model has been used in other research to explore the intensity of cultivation in multiple time periods in Germany (Bogaard et al. 2016; Styring et al. 2017; Hamerow et al. 2022; Hamerow et al. in press), as well as Iron age, Roman and medieval England (Hamerow et al. 2020; Lodwick 2023) and Iron Age France (Alagich et al. 2018).

### Model 2: high- versus low-input farming (Asturias, Evvia, Provence and Morocco)

Arable field surveys in Morocco provided data from oases and rain-fed fields in a semi-arid region, allowing for the construction of model 2 (Bogaard et al. 2018). This model incorporated data from model 1 (Haute Provence and Asturias) and from Evvia in Greece (Jones et al. 1999, 2000). Model 2 uses data from three different locations to represent high-intensity cultivation (Moroccan oases, Evvian gardens and Asturian plots) and low-intensity regimes (Moroccan rain-fed terraces, Evvian fields and Haute Provence fields). The model places the emphasis on fertility rather than soil disturbance, since fertility-related traits successfully separated high- and low-intensity regimes and the inclusion of disturbance attributes did not improve this separation further. The functional traits used in the model are canopy height, canopy diameter, leaf area per node/leaf thickness, and mean specific leaf area. This model has been applied to semi-arid locations, in particular in western Asia (Bogaard et al. 2018; Green et al. 2018; Diffey et al. 2020; Stroud et al. 2021; Maltas et al. 2022).

### Model 3: high versus low disturbance (Highgrove and Laxton)

Model 3 uses data from two locations within the UK to distinguish levels of soil disturbance irrespective of fertility. The model uses modern botanical survey data from Laxton in Nottinghamshire and Highgrove’s Duchy Home Farm in Gloucestershire. The Laxton data include surveys of unploughed but periodically grazed and cut hay meadow areas on the edges of the open fields (called ‘sykes’), as well as unsprayed edges of strip cereal fields and fallow fields, managed within an open-field system, while the Highgrove data are from cereal fields cultivated under a different rotation system (see Bogaard et al. 2022 for full details). The combination of these data provides a comparison of highly disturbed arable fields with unploughed meadow areas, creating a model with which to explore disturbance. The created model used the traits of vegetative propagation and flowering period. This model has been used to investigate levels of disturbance expected under mouldboard plough tillage, through comparison with experimental ridge-and-furrow fields at Lorsch, Germany, and to assess how arable disturbance levels developed through the English medieval period, when the mouldboard plough is hypothesised to have become widely used (Bogaard et al. 2022).

## Data collection, functional traits and data quality

The data for the models come from botanical surveys of modern fields; in each field the weed species present within one metre square quadrats were recorded. These surveys were conducted in five to ten 1 m^2^ quadrats along a transect from one end of the field to the other. For use in the models and comparison with archaeobotanical data, the botanical survey data were converted to species’ presence/absence per field. The average score of the functional traits per field was then calculated as the sum of the attribute value for the species divided by the number of species in the field (see Bogaard et al. 2016, 2018, 2022 for full details).

The averaged functional trait data per survey field is published with this paper and is required to run the models: in the case of the R package, these data are written into the functions (ESM 1). The published functional trait data of 928 archaeobotanically relevant species (see Hodgson et al. 2023) are also written into the R package.

The values of five functional traits are provided for all 928 species: SLA, ARNODE, LOGCANH, LOGCAND and VEGPROP is accessible via the R package as well as Hodgson et al. (2023) (see Table 1 for full details). An additional functional trait required for model 1 and model 3, FLOWPER, is not provided and requires users to obtain these data from relevant Floras that cover their study region. The trait values are from multiple specimens of that species from different geographic locations. To capture the species’ potential, rather than an individual plant’s performance, every effort was made to ensure that the plants collected for trait measurement were both mature and from optimal field conditions.

**Table 1:** The six functional traits used within the different models, and whether the data is provided by Hodgson et al. (2023)

Trait code	Meaning	Calculation	Provided?	Model 1	Model 2	Model 3
SLA	Specific leaf area	Mean of leaf area (mm^2^) per unit of dry leaf weight (mg)	Yes	Yes	Yes	
ARNODE	Leaf area per node/ leaf thickness	Mean of leaf area (mm^2^) per node/ leaf thickness (mm)	Yes	Yes	Yes	
LOGCANH	Log of maximum canopy height (cm)	Maximum of: measured canopy height divided by measured plant height, multiplied by max plant height	Yes	Yes	Yes	
LOGCAND	Log of maximum canopy diameter (cm)	Maximum	Yes	Yes	Yes	
FLOWPER	Flowering period	Length in months	No	Yes		Yes
VEGPROP	Vegetative propagation	Yes or no	Yes			Yes

The status of the archaeobotanical data to be compared against one of the models—as a reliable representation of crops and weeds—clearly shapes the results. The most robust results are obtained by using archaeobotanical data from contexts where the weed seeds are directly attributable as arable weeds i.e. storage contexts (e.g. Green et al. 2018; Diffey et al. 2020). Material from other contexts can be used but this requires data cleaning to remove any items which are not arable weed species. In secondary or tertiary deposits distinguishing the arable weeds from other taxa (i.e. inputs from dung burning, edible wild taxa etc.) is necessary (Stroud et al. 2021) and we recommend using additional techniques such as correspondence analysis and/or discriminant analysis-based crop processing identification methods (e.g. R package CropPro, Stroud et al. 2023a) to understand which taxa are likely to be arable species. Additionally, it is recommended that an archaeobotanical sample contains a minimum of 10 weed seeds (identified more or less to species), and ideally far more than that to be representative of the fields from which they derive (e.g. Diffey et al. 2020). Research has also shown that archaeobotanical samples with a low diversity of species can yield erratic results; as such it is advisable to have at least three species per sample or to examine changes in the results when samples with fewer than 3 species are included and when they are not (e.g. Alagich et al. 2018).

## Functionality of the R package

The R package WeedEco allows users to organise raw archaeobotanical data and then conduct linear discriminant analysis to classify those data against the supplied modern models. WeedEco also has functions to produce plots of the output of the linear discriminant analysis. WeedEco can be broken up into three different groups of functions: data organisation, classification, and visualisation. The package can be downloaded into R from GitHub^1^ using the devtools package by Wickham et al. (2022). The package WeedEco can be manually downloaded from the WeedEco GitHub account or the code below can be used to download it within R using the devtools package’s function install_github:

install_github(“WeedEco/WeedEco”).

### Data organisation

The functional trait database is organised by species, with each species designated a four-three species code (henceforth called species code). Where possible a species code is made up of the first four letters of the genus name and the first three letters of the specific epithet. There are exceptions to this rule such as when a code is not unique (e.g. ***Gali****nsoga* ***par****viflora* and ***Gali****um* ***par****isiense*), or the species does not have a genus name four letters long. For taxa that have an identical four-three code, commonly the next letter is used. It should be noted that species with genus names less than four letters long are separated in the code with a “_”; e.g. poa_ann for *Poa annua*. These four-three species codes are used to extract functional data from the database by the R package WeedEco. It is therefore essential that the species codes used are the correct codes for the species required. The four-three codes of the species currently available in the database, as well as their corresponding Flora Europaea and World flora online numbers can be found in ESM 2 or accessed within R using the *weed_data* function (Tutin 1964–1993; WFO 2023). Users are advised to check with species synonyms as the four_three codes relate to the taxonomic consensus at the time the data was collected. When comparing synonyms, it is recommended to verify that both the authority and the name are a match. The World flora Online (WFO 2023) serves as a valuable resource for cross-referencing and verifying synonyms. If taxa (and four_three codes) are included in the archaeobotanical dataset that are not included in the functional trait database, R will exclude them from the analysis (and an error message will be produced).

The function *wdata_org* organises a raw archaeobotanical spreadsheet into the format required for linear discriminant analysis. It changes the archaeobotanical data into presence/absence data and then extracts the functional trait values, based on the species codes, of the species within each sample. Finally, the averaged functional attribute values for each trait per sample is calculated, returning a data frame^2^ which is suitable for use within the classification functions of the package.

WeedEco’s two other data organisational functions are *weed_data* and *ave_wdata.* The function *weed_data* allows users to extract functional trait data based on the entered species codes and is useful if the data are required for alternative uses or validity checking. *ave_wdata* was created to deal with occurrences of specimens which cannot be identified to species, allowing users to average the functional trait data of a number of species to form a composite value. If the genus has limited species, or the specimen is one of only a few species, then it is possible to average the multiple species’ trait data to produce trait values which can be used in *wdata_org*. Users are recommended to be cautious in averaging more than three or four species, or in averaging values that are very different.

### Classification

WeedEco provides a function to conduct linear discriminant analysis called *wmodel.LDA*. It uses in part the MASS package’s *lda* function, but wraps it, allowing for the comparison of the inputted archaeobotanical data against one of the three included modern crop regime models. This makes use simple with the only inputs needed being the dataset produced from *wdata_org*, as well as instructions on which model is required. If the output of *wdata_org* is not used then the function requires specific column names and order for the averaged functional trait data for each archaeobotanical sample (SLA, ARNODE, LOGCANH, LOGCAND, VEGPROP, FLOWPER).

*wmodel.LDA* conducts discriminant analysis on the selected comparative modern model: such data are stored within the R package but are also included in ESM 1. *wmodel.LDA* creates a discriminant model that is used to classify the entered archaeobotanical data as either regime 1 or regime 2, with interpretation dependent on the selected model (column called Class). *wmodel.LDA* also provides the posterior probability of each archaeobotanical sample falling within group 1 or group 2 (Prob.1 and Prob.2). The samples’ linear discriminant scores (LD1) are also calculated, and they are later used within the plotting functions. The function provides both standardised and un-standardised data, as well as the unstandardised centroid values for group 1 and group 2 (for further detail on the linear discriminant analysis procedure see MASS package help file, or Venables and Ripley 2002). Further information on the returned data is provided in the WeedEco help file (Stroud et al. 2023b).

### Visualisation

**Fig. 1:**
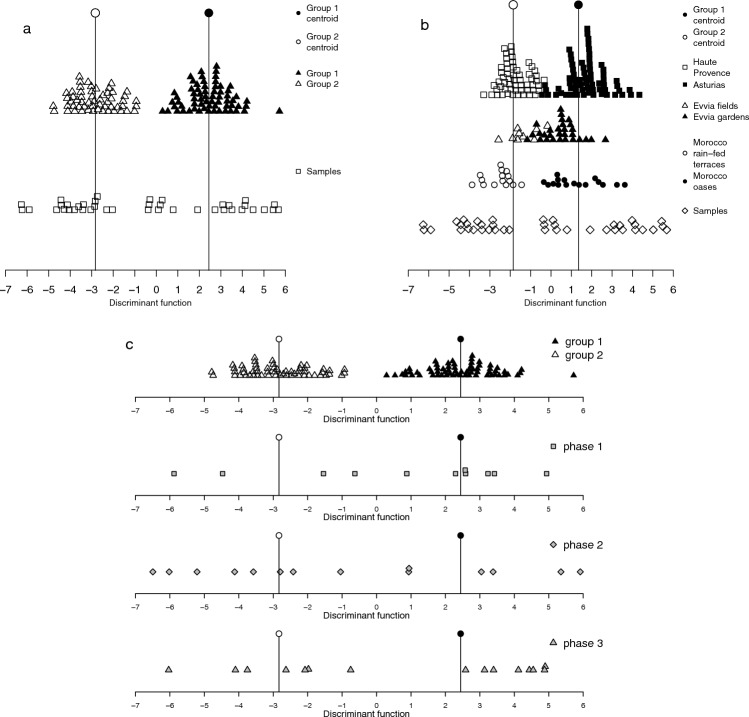
The three plotting options from WeedEco: **a** *wplot_basic* showing random data against model 1; **b** *wplot_geog* showing random data against model 2; **c** *wplot_phase* showing random data plotted by phase against model 1

The final group of functions provide options for plotting the output of *wmodel.LDA*. The three options vary in the amount of detail shown of the classifying model used. For example, *wplot_basic* plots the archaeobotanical samples’ discriminant score against the model’s samples and centroids (Fig. 1a). *wplot_geog* separates out the model’s samples into their different geographical locations—this is particularly relevant for model 2 and model 3 which have modern data from multiple geographical locations within the same model group, and *wplot_geog* allows all the locations contributing to group 1 or group 2 to be displayed (Fig. 1b). The final plotting option, *wplot_phase*, produces a stacked graph of up to five subplots allowing the display of multiple sites or different phases (Fig. 1c). *wplot_phase* is more complex than the other plotting functions which only require the model number and the column with the LDA data from the *wdata.LDA* output; *wplot_phase* requires an additional grouping variable.

The plotting functions use the Beeswarm package’s function *swarmy*, and some of that function’s arguments can be used to modify the arrangement and order of the points (compact and priority) (see Eklund and Trimble 2021). Further modification is possible of the axes, limits, symbols, colour, and legend location (see below and Stroud at al. (2023b) for more detail).

## The case study site of medieval Stafford

### Model 1

Archaeobotanical data from the early medieval site of Stafford was analysed by Hamerow et al. (2020) and Bogaard et al. (2022) to understand the evolving nature of the crop production system and to assess a hypothesised trend towards extensification (expansion through low-input farming) during this period of agronomic reorganisation. Such analysis was conducted using SPSS and plotted in excel, so here we re-analysed the Stafford data using WeedEco to demonstrate the utility of the R package. The archaeobotanical samples come from three excavations in the centre of Stafford: St Mary’s Grove, Bath Street and Tipping Street South (Carver 2010; Dodd et al. 2014; Hamerow et al. 2020; McKerracher et al. 2023). The data spans four broad phases of occupation from the late 9th to 16th centuries. The raw data derive from original archaeobotanical analyses by Moffett (1987) and Druce (2014); the phasing used in this paper was devised by the FeedSax project (Hamerow et al. 2020). The dataset contained only charred items.

**Fig. 2:**
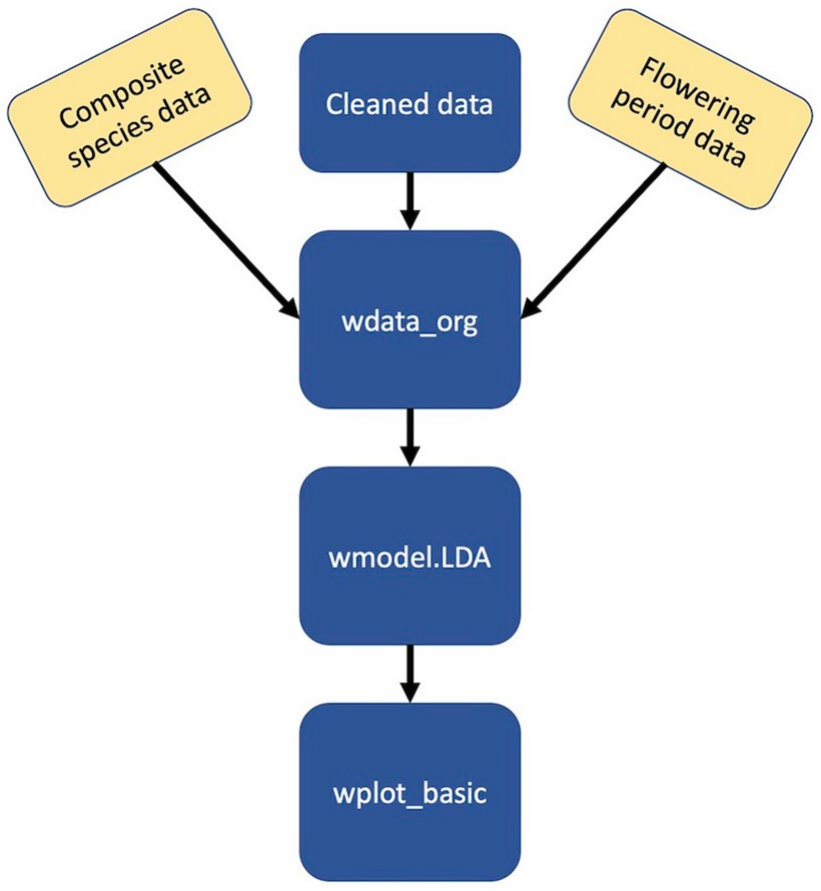
A basic flow chart of the main steps required using the main WeedEco functions; yellow boxes are not always required

The use of WeedEco revolves around three functions (Fig. 2), however a large amount of data cleaning is required before these functions can be used. Each step required for the Stafford data is described in detail below. It should be noted that this does not include the use of model 2, due to its geographical/environmental unsuitability for Stafford. An R script containing the step-by-step processes involved to analyse and plot the Stafford data using WeedEco is included as ESM 3.

The raw archaeobotanical data from Stafford, a total of 46 samples, was ‘cleaned’: any crop species or non-arable taxa such as woody taxa unlikely to set seed in arable conditions and collected/foraged taxa were removed. Any tentative identification—for example cf. identifications—were either removed or added to positively identified tallies of that species, as appropriate. Taxa that could not be identified to species were either excluded or, if thought to be one of a small number of species with similar functional trait values, included as a composite taxon, with occurrences summed into one row (Table 2). A column called species.codes was added to the datasheet which included the species code of each taxon obtained from ESM 2 or using *weed_data* (ESM 3, code line 28) (Table 2, column 1). For composite taxa, a species code was constructed using something unique and memorable, making sure it was not an already existing four_three species code. If a species is not in the supplied spreadsheet, this indicates that full functional trait data is not available for it: the species either needs to be removed or functional trait data obtained from elsewhere. The only species which did not have trait data, *Carex flava*, was retained, as trait data was obtained from an alternative source (see below). Only archaeobotanical samples which contain at least ten seeds of weed species in the final cleaned dataset were kept, resulting in a total of 45 samples with a total of 80 taxa. The final cleaned dataset is shown in ESM 4.

**Table 2:** The format of the raw dataset from Stafford showing the first eight samples and first 12 taxa; full dataset in ESM 4

Species.codes	Taxa	11	115	118	203	204	205	221
agrogit	*Agrostemma githago*	6		6	2	4	9	
anetgra	*Anethum graveolens*							
anthcot	*Anthemis cotula*			18				
arrhels	*Arrhenatherum elatius*		1					
**avenstfa**	***Avena sterilis/A. fatua***							
brasnig	*Brassica nigra*					4		
**bromhose**	***Bromus secalinus/B. hordeaceus***		1					29
buglarv	*Lithospermum arvense*							
buplrot	*Bupleurum rotundifolium*							
careflv	*Carex flava*							
carenig	*Carex nigra*							
carepna	*Carex panicea*							
centcya	*Centaurea cyanus*							
centnig	*Centaurea nigra*							
chenalb	*Chenopodium album*	6			8	76	220	
chenmur	*Chenopodium murale*							
chryseg	*Glebionis segetum*	4		8				
conimac	*Conium maculatum*							
cynocri	*Cynosurus cristatus*							
dauccar	*Daucus carota*							
**eleounpa**	***Eleocharis uniglumis/E. palustris***							

The rows in bold are composite species and the first column shows the species codes for each taxon obtained from ESM 2 or the function *weed_data*

In addition to the cleaned raw dataset, a spreadsheet detailing the flowering periods of each of the 80 taxa in the Stafford dataset is required. Flowering period (FLOWPER) data are not provided within the trait dataset due to geographical differences, so such data needs to be collated from relevant Floras and imported into R. A spreadsheet was constructed using flowering data from a UK Flora (Clapham et al. 1987) supplemented by data from a German Flora (Rothmaler 1995), containing a column of species codes and a column with the flowering duration in months (Table 3, ESM 5). Note that FLOWPER is only needed for model 1 and model 3. For composite taxa an averaged flowering period of the multiple species was entered.

**Table 3:** The format of the second spreadsheet required: flowering periods

Species.codes	FLOWPER
agrogit	2
anetgra	3
anthcot	5
arrhels	2
avenstfa	3
brasnig	4
bromhose	2
buglarv	4
buplrot	2
careflv	5
carenig	2
carepna	2

The first 12 species from Stafford are shown with composite species in bold, species missing trait data underlined

For the Stafford dataset composite taxa were created when an item could have derived from one of two or three species. Before constructing the averaged functional traits of the composite species, *weed_data* was used to examine the species, confirming that their functional traits were not widely divergent (code line 28). How divergent is too divergent is subjective, however it should be noted that averaging extremely different values will produce a meaningless value which is potentially detrimental to the analysis. Therefore, species with very different growing requirements or functional traits should be removed, or have the divergent values removed (i.e. replaced with NA—see below regarding VEGPROP).

**Fig. 3:**
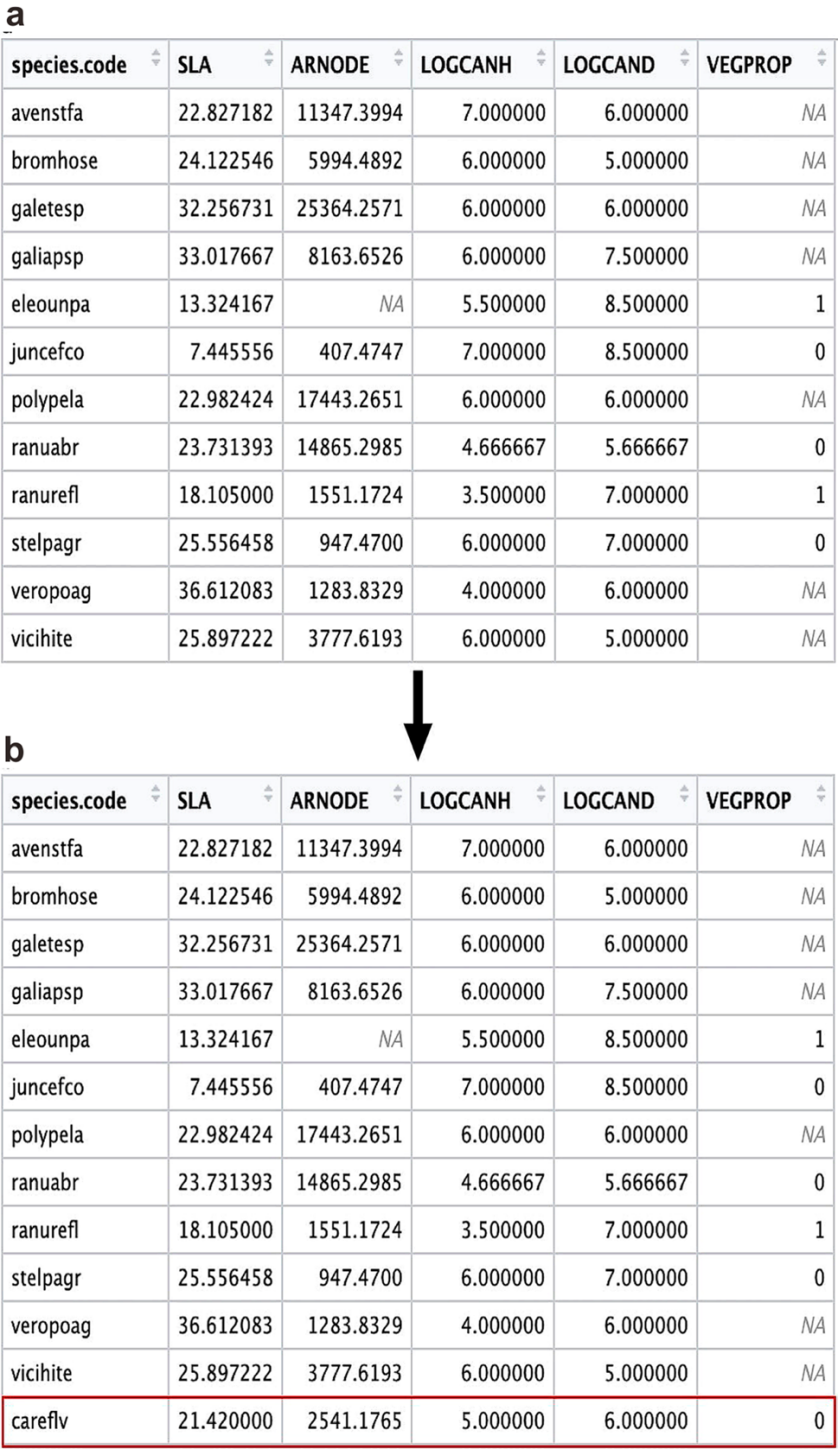
**a** The results of averaging the composite taxa trait values using *ave_wdata*; **b** the modified *ave_wdata* output with the *Carex flava* data added to the last row

To obtain the composite taxa’s functional trait data the function *ave_wdata* was used. The Stafford data contained 12 composite taxa (Table 4) and *ave_wdata* was used to average the different species’ functional trait values, creating an averaged SLA, ARNODE, LOGCANH, LOGCAND and VEGPROP for each composite taxon. For example, using ave_wdata the functional trait values of the species *Avena sterilis* (avenste), and *Avena fatua* (avenfat) were averaged together to create the composite taxon avenstfa. This was done for all composite species to create a data frame containing those taxa and their averaged functional trait data (code line 36–49; Fig. 3a).

**Table 4:** The new species codes for the composite taxa from Stafford and their included species

Species code	Species 1	Species 2	Species 3
avenstfa	avenste	avenfat	
bromhose	aromsec	bromhor	
galetesp	galetet	galespe	
eleounpa	eleouni	eleopal	
galiapsp	galiapa	galispu	
juncefco	Junceff	junccon	
polypela	polyper	polylap	
ranuabr	ranuacr	ranubul	ranurep
ranurefl	ranurpt	ranufla	
stelpagr	stelplu	stelgra	
veropoag	veropol	veroagr	
vicihite	vicitet	vicihir	

*Carex flava* was identified within the Stafford assemblages, but trait data are not available for this species within the released dataset. There are two options when this occurs: exclude this species during data cleaning or manually add data from alternative sources. As we have data for this species from alternative sources it was added to the composite taxa data frame as a new row (code line 46; Fig. 3b). The data from *Carex flava* came from partial trait data for that species already collected as well as data from alternative sources (ESM 6).

At this point, three datasets have been created: the cleaned raw Stafford data, the composite taxa trait values (including the additional *Carex flava* data), and the flowering period data for all 80 of Stafford’s weed taxa. The cleaned data and flowering period datasets can be created outside R, while the composite taxa trait values require R and the functions within the WeedEco package.

**Fig. 4:**
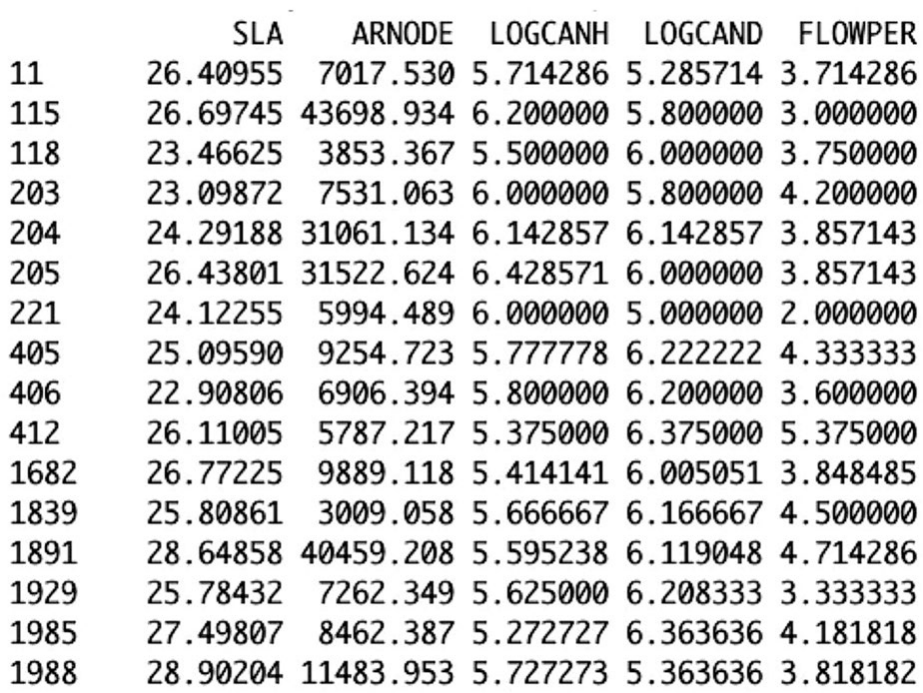
Part of the output of *wdata_org* showing the averaged functional trait data of the species within the archaeobotanical samples from Stafford; this is output of *wdata_org* when the model assigned as model 1

The next steps within R combine the three created datasets in the correct format for linear discriminant analysis. The *wdata_org* function combines the three datasets, FLOWPER, composite taxa and the cleaned dataset, to produce a new data frame that contains the averaged functional trait values of the taxa within each archaeobotanical sample from Stafford (Fig. 4). Each species code in the Stafford data is used by *wdata_org* to extract the functional trait data for the species from the trait database. It is then averaged with the other species trait values within that sample. The resultant data frame produced was saved as an R object for use when conducting discriminant analysis (code line 56).

**Fig. 5:**
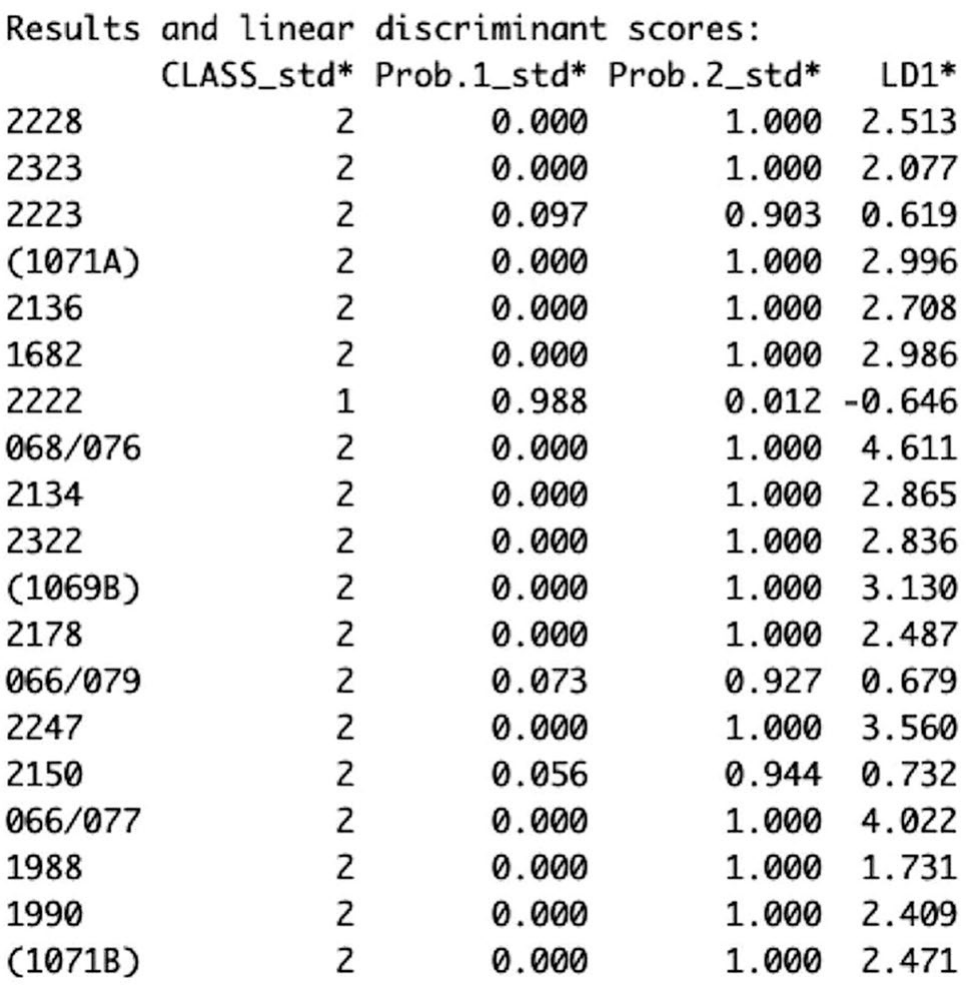
Part of the console output of *wdata.LDA* showing the classifications (CLASS_std*), posterior probabilities (Prob.1_std*, Prob.2_std*) and linear discriminant function 1 score (LD1*)

As explained above, WeedEco’s linear discriminant analysis function *wdata.LDA* takes the output of *wdata_org* and conducts discriminant analysis—in particular the classification stage on the averaged trait values of the samples. The results of running the Stafford data though *wdata.LDA* is shown in Fig. 5, which gives the classification (group 1 vs. group 2) (Class_std*), the posterior probability of the sample being in group 1 (Prob.1_std*) or 2 (Prob.2_std*), and the linear discriminant scores (LD1*) of each archaeobotanical sample. The full results of the analysis can be viewed when the *wdata.LDA* is saved as a data frame and will show both standardised and unstandardised LDA scores, classifications and probabilities (see the WeedEco help document or Venables and Ripley (2002) for full details). The unstandardised linear discriminant scores (LD1*) are used in the WeedEco plotting functions. Interpretation of the results is done visually by plotting the linear discriminant scores against the discriminant scores of the model’s samples or against the model’s centroids. The results of the Stafford discriminant analysis were saved as a data frame to be used in the plotting functions (code line 59).

The different plotting functions in WeedEco provide a range of options as explained above and shown in Fig. 1. *wplot_basic* was used for the Stafford data and shows that the majority of the Stafford samples plot around the group 2 centroid, indicating that the samples more closely resemble the Haute Provence data, indicating low-input cultivation with relatively low fertility and disturbance, and consistent with the hypothesis of extensification (code line 62). To present the data in the same way as Hamerow et al. (2020, Fig. 6) *wplot_phase* was used, with the data separated by four broad phases (Fig. 7). A column delineating the phasing of all the samples was added to the output of *wdata.LDA* (code line 66, 67, ESM 7). Each phase was then graphed on separate subgraphs, each with different colours or symbols (gcol, gbg and gpch, code line 69). By separating the results into broad phases, change over time can be examined, with the Stafford data showing that the earliest phase has variable scores while, from the mid tenth century onwards, the samples are more regularly around the centroid of group 2. This indicates a trend towards extensification over time at Stafford, with little to no manuring or hand weeding from the mid tenth century onwards (see Hamerow et al. 2020 for full details).

**Fig. 6:**
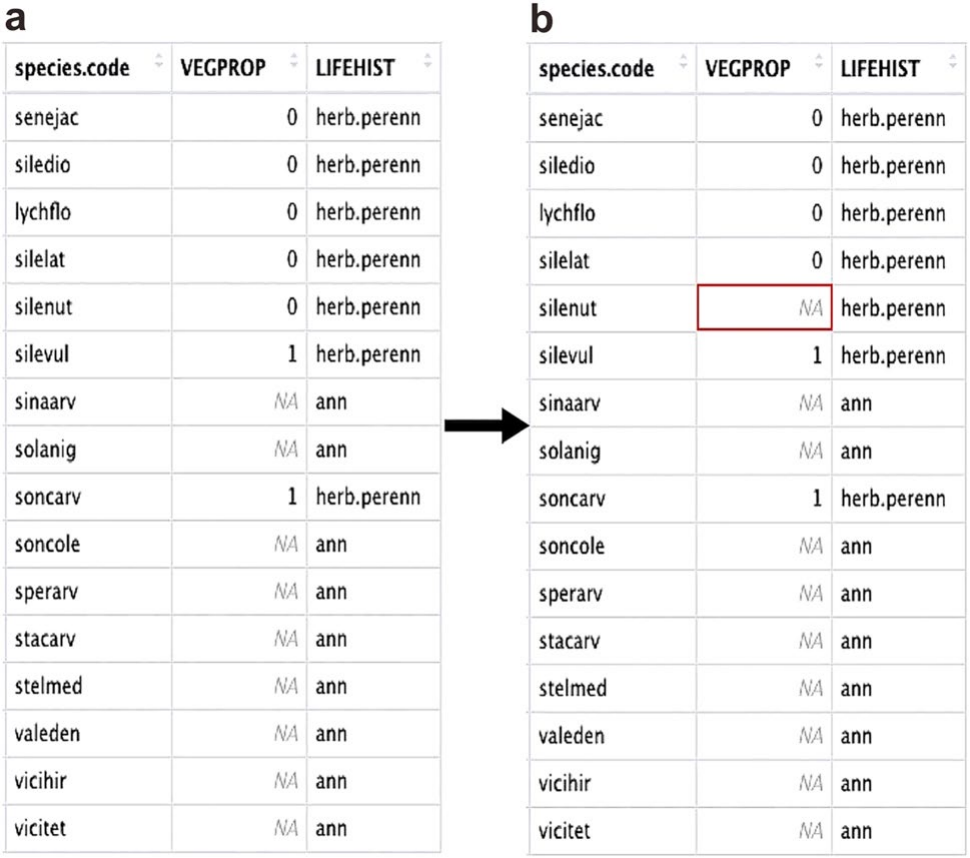
A subset of the results of the function *weed_data* (**a**) and then the changed entry for *silenut* in (**b**)

**Fig. 7:**
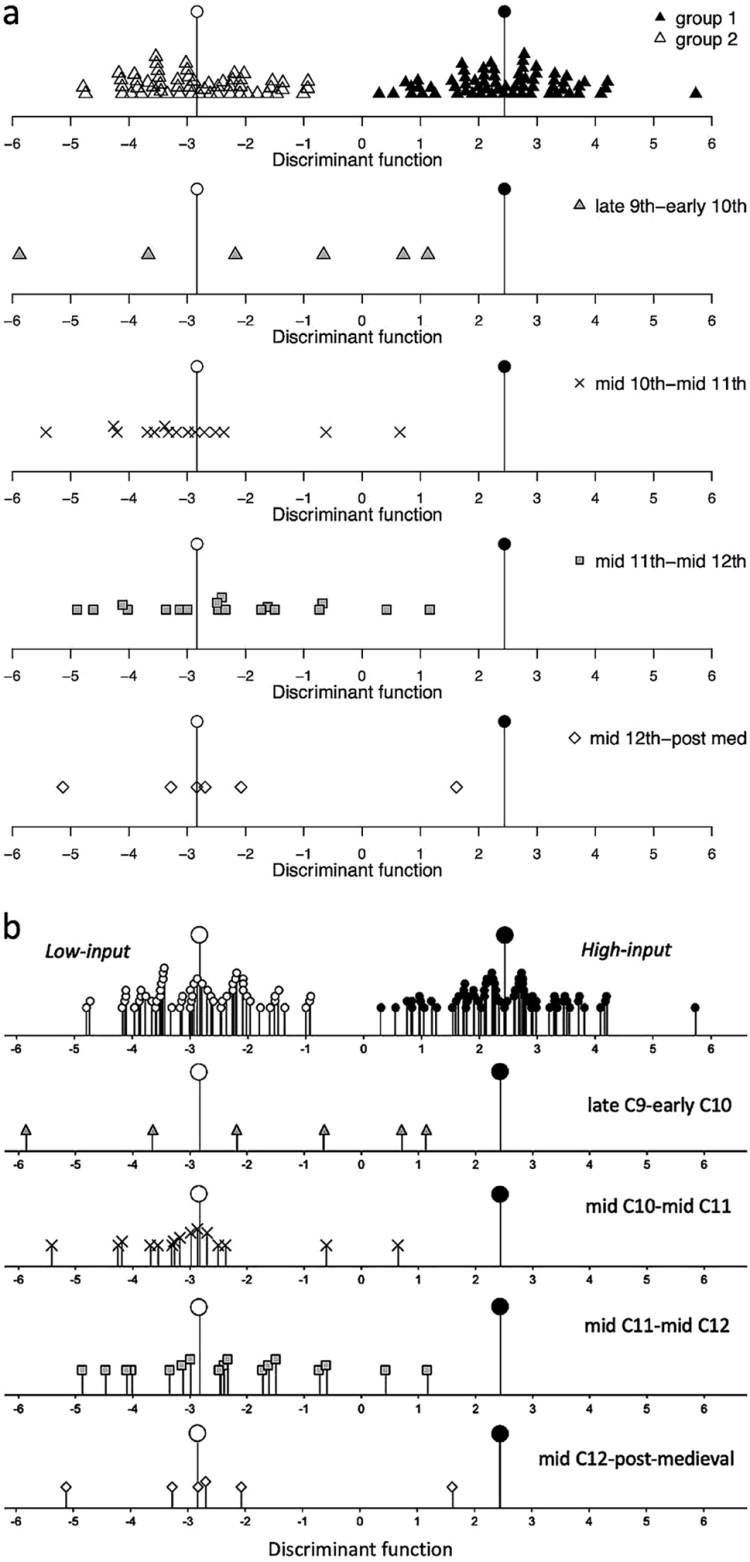
**a** The output of *wplot_phase* using the Stafford data and model 1; **b** Fig. 7 from Hamerow et al. (2020) showing the results of the analysis in SPSS (plotted in Excel) using the same data

### Models 2 and 3

The WeedEco functions can also be used to compare archaeobotanical data against model 2 and model 3, with only a few differences in usage. Model 2 *wdata_org* only requires the raw archaeobotanical datasheet and the composite taxa data frame. All other functions are used in the same way as model 1, except for entering ‘model 2’ whenever the model number is required.

Model 3 differs from the two other models as it only uses two functional attributes: FLOWPER and VEGPROP. The data cleaning steps required for model 3 are the same as those described above for model 1 and, if already completed, cleaned data from model 1 can feed into model 3. There are however differences in usage of WeedEco between model 1 and model 3, due to the functional trait VEGPROP, which can require some extra steps.

When averaging composite taxa *ave_wdata* is again used to produce the dataset, but for the Stafford taxa there were uncertainties as to the VEGPROP value of composite taxa ranuabr and ranurefl. It was not certain that the items within those two composite taxa necessarily came from perennial species, nor that they displayed vegetative propagation, so the VEGPROP value of these two composite taxa were modified to show no data, excluding them from any VEGPROP calculations (code line 78, 79).

It is recommended that when using model 3 users confirm their understanding of the life history of each species and whether the typical life history of that species within their geographical region is reflected. Using the *weed_data* function, species found within the Stafford data were entered and the trait “VEGPROP” selected (code line 82). This produced a table of the vegetative propagation ability of the entered species, as well their life history (Fig. 6a). In the Stafford data there was uncertainty as to whether *Silene nutans* (silenut) should be counted within the VEGPROP calculations—in the dataset it shows as a perennial and therefore a 0 within the VEGPROP column (Fig. 6b). To remove it from any averaged calculation of VEGPROP the silenut’s VEGPROP value was changed from a 0 to an NA (code line 85). Note that it is the VEGPROP value, not the LIFEHIST value, that is changed as it is the VEGPROP trait which is directly used in model 3. The altered data frame was then entered into the *wdata_org* function using the argument vg_pr, along with the other required datasets, raw data, composite taxa and flowering period, as well as the other associated instructions (‘model 3’ etc.) (code line 88).

**Fig. 8:**
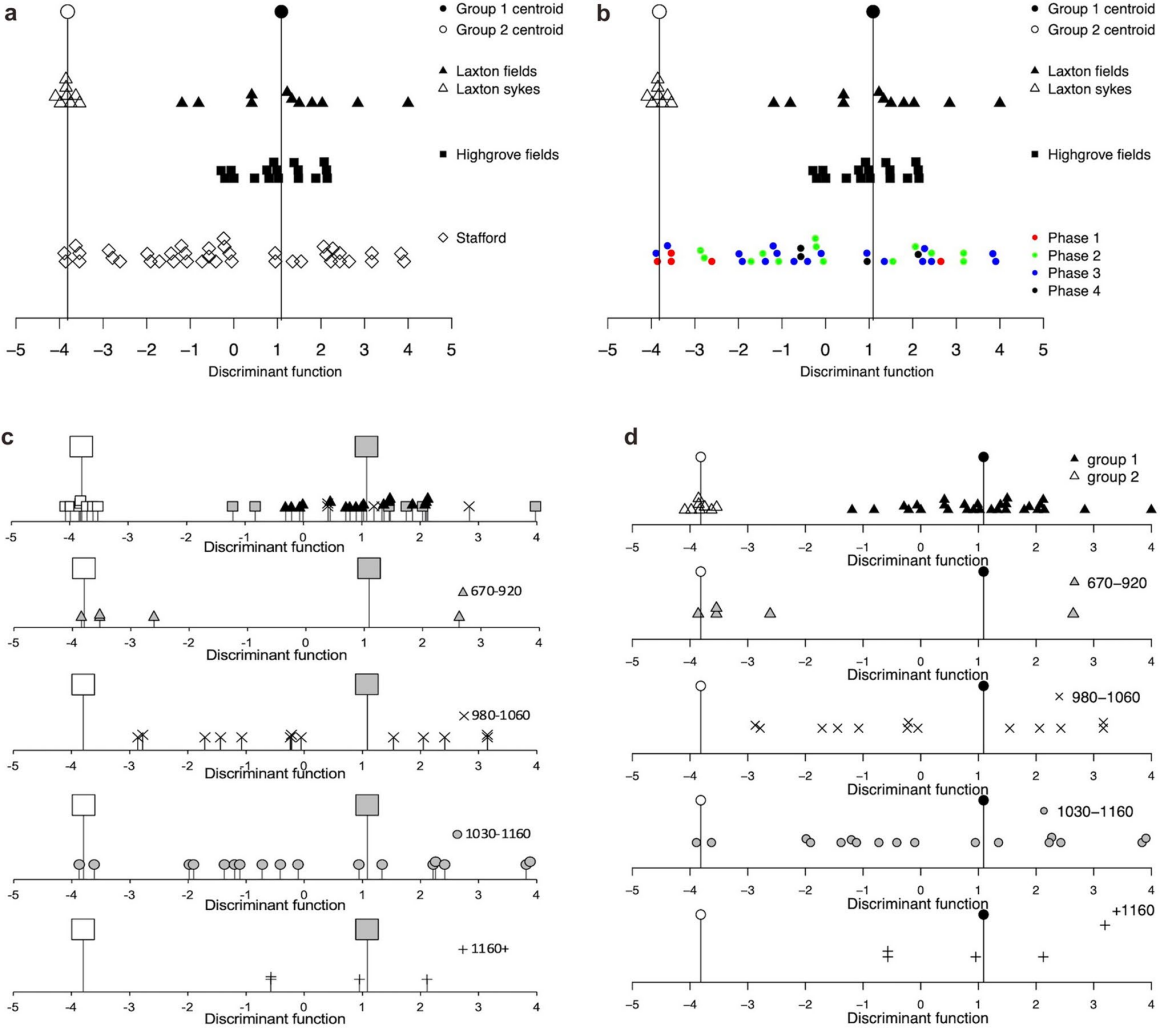
**a** The Stafford data classified against model 3, plotted using *wplot_geog*; **b** the results of modifying colour and symbol augments in *wplot_geog*; **c** Fig. 14 from Bogaard et al. (2022) created in Excel from data produced in SPSS compared to **d** the Stafford data plotted using *wplot_phase*

The output of *wdata_org* was then analysed with *wmodel.LDA* using the model argument of model 3 (code line 88). This produced the linear discriminant result, which, as with model 1, can then be plotted using one of the functions. For example, *wplot_geog* produces Fig. 8a which can be further modified by, for example, changing the symbols, colour, and axis limit. One helpful aspect of the plotting functions is that the colours and symbols of different subsets of the data can be changed—allowing the visualisation of multiple phases on one graph (Fig. 8b). However, to more easily see change through time *wplot_phase* allows comparison of separate phases within sub graphs (see Fig. 8c). By calling on the newly created phase column each phase is plotted as a subplot. To make the symbols the same as Bogaard et al. (2022) (Fig. 8d), the gpch, gcol and gbg arguments need to be modified from the defaults (code line 122, 123) (Fig. 8c).

Comparing the Stafford data to model 3 shows a tendency through time for the samples to occur more towards centroid 1, the high disturbance end of the graph, indicating that the samples increasingly resemble the conditions of the modern arable fields. Figure 8c shows that from the 12th century onwards no samples resemble the undisturbed model samples. Before the 12th century there are samples which are similar to the undisturbed model samples which may represent the arable-grassland interface or the cultivation of land normally used as pasture (Bogaard et al. 2022). Consistent and comprehensive disturbance seen in the 12th century samples has been interpreted as the more systematic use of the mouldboard plough (Bogaard et al. 2022).

## Discussion

The R package WeedEco and its models allow users to investigate the farming regime represented by archaeobotanical samples. As shown above, the package provides mechanisms to organise, analyse and visualise archaeobotanical data in reference to three present-day models. The example provided using the Stafford data demonstrates the use of the package’s functions and data.

Previously published functional weed ecology analysis of archaeobotanical weed seeds has been conducted in SPSS but to enable accessibility, an equivalent process in R was perceived as important when these data were released. In the transition from SPSS to R small differences were noted in how each statistical program ran linear discriminant analysis. The primary difference is that, due to being purely arbitrary, the positive and negative signs for group 1 and group 2 were reversed between SPSS and R. To make the graphs similar to the published output from SPSS, the R package WeedEco formally made all group 1 linear discriminant values positive and all group 2 linear discriminant values negative. This should be noted should the raw model data be run in alternative statistical programs. Furthermore, users should indicate if R is not used for the linear discriminant analysis due to these slight differences. This does not prevent alternative statistical programs being used but, for ease of comparison between different archaeobotanists’ results, explicitly stating what has been used in the methods is best practice.

The trait data used in the R package was actively collected up until 2021. Changes to the underlying trait data will occur in the future as more accessions are recorded in the field. Furthermore, as models are updated and new models are developed, the R package will be updated to reflect this. Any changes to the underlying trait data used will be noted in future updates of the R package with new versions published on the Oxford University Research archive. Due to the potential multiple versions of these data, it is strongly recommended that the version of the R package, R, RStudio, and the trait dataset are explicitly stated within the method section of outputs to facilitate reproducibility. To cite the use of the data, models and R package described in this article we suggest including a paragraph referencing all the components. Again, using the Stafford dataset as an example, a paragraph like the one below should be included:

The analysis followed the procedure described in Stroud et al. (this paper). The R package WeedEco, version 1.0.0, was used to extract the functional trait values of the Stafford weed species from the trait database, version 1 (Hodgson et al. 2023, Stroud et al. (this paper), Stroud et al. 2023b). The Stafford samples were classified using the discriminant analysis function within WeedEco against model 1 and 3 (see Bogaard et al. (2016) and Bogaard et al. (2022) respectively for model details). R version 4.2.2, and RStudio version 2022.07.02, were used.

## Conclusions

The released model data and R package, in conjunction with the functional trait data (Hodgson et al. 2023), provide a resource allowing archaeobotanists to investigate the crop husbandry regimes and practices represented within the weed flora of crops. Model 1 and model 2 provide an insight into the degree of high or low input cultivation practiced in the past—regarding fertility and disturbance for model 1 and fertility only for model 2. Model 3 allows users to disentangle the effects of fertility and disturbance levels, by examining the nature of disturbance within crop fields, irrespective of variations in fertility. The R package WeedEco allows users to access the trait data and, through discriminant analysis, to compare their archaeobotanical samples with the model(s) of their choice.

### Supplementary Information

Below is the link to the electronic supplementary material.

Supplementary material 1 (XLSX 26.6 kb)

Supplementary material 2 (XLSX 140.8 kb)

Supplementary material 3 (R 9.0 kb)

Supplementary material 4 (XLSX 16.3 kb)

Supplementary material 5 (XLSX 12.7 kb)

Supplementary material 6 (XLSX 10.4 kb)

Supplementary material 7 (XLSX 11.5 kb)

## Footnotes

1 The authors aim to submit the package to CRAN in the near future. Currently the development version of the package is available on GitHub.

2 R uses a wide range of data formats. The package WeedEco was written assuming the data will be entered as a data frame—which is a table-like two-dimensional array in which there are columns with an equal number of rows.
